# Evaluating outcomes of same-day discharge after catheter ablation for atrial fibrillation in a real-world cohort

**DOI:** 10.1016/j.hroo.2021.07.001

**Published:** 2021-07-14

**Authors:** Michael E. Field, Laura Goldstein, Kevin Corriveau, Rahul Khanna, Xiaozhou Fan, Michael R. Gold

**Affiliations:** ∗ Medical University of South Carolina, Charleston, South Carolina; † Medical Devices Franchise Health Economics and Market Access, Johnson & Johnson, Irvine, California; ‡ Medical Device Epidemiology and Real-World Data Sciences, Johnson and Johnson, New Brunswick, New Jersey

**Keywords:** Atrial fibrillation, Catheter ablation, Efficacy, Safety, Same-day discharge

## Abstract

**Background:**

As same-day discharge (SDD) after catheter ablation (CA) for atrial fibrillation (AF) is increasingly utilized, it is important to further investigate this approach.

**Objective:**

To investigate the safety and efficacy of SDD after CA for AF in a large nationwide administrative sample.

**Methods:**

The IBM MarketScan Commercial Claims and Encounters database was used to identify adult patients under 65 years undergoing CA for AF (2016–2020). Eligible patients were indexed to date of first CA and classified into SDD or overnight stay (ONS) groups based on length of service. A 1:3 propensity score matching was used to create comparable SDD:ONS samples. Study outcomes were CA-related complications within 30 days after index procedure and AF recurrence within 1 year. Cox proportional hazards models were estimated for outcome comparison.

**Results:**

In the postmatch 30-day cohort, there were 1610 SDD and 4637 ONS patients with mean age 56.1 (± 7.6) years. There was no significant difference in composite 30-day postprocedural complication rate between SDD and ONS groups (2.7% vs 2.8%, respectively; *P* = .884). The most common complications were cerebrovascular events (0.7% vs 0.7%; *P* = .948), vascular access events (0.6% vs 0.6%; *P* = .935), and pericardial complications (0.6% vs 0.5%; *P* = .921). Further, no significant difference in composite AF recurrence rate at 1 year was observed among SDD and ONS groups (10.2% vs 8.8%; hazard ratio = 1.167; 95% confidence interval 0.935–1.455; *P* = .172).

**Conclusion:**

In a large, propensity-matched, real-world sample, SDD appears to be safe and have similar outcomes compared with overnight observation following CA for AF.

**Key Findings**

▪ To address the increasing demand for catheter ablation of atrial fibrillation, same-day discharge (SDD) protocols may help reduce healthcare resource utilization and increase patient satisfaction.
▪ SDD after atrial fibrillation ablation has been shown to be safe and effective based on small multicenter and single-center studies.
▪ In our analysis of a large, real-world administrative claims dataset, the rate of postdischarge complications and procedure efficacy were similar in patients undergoing atrial fibrillation ablation with SDD compared to overnight stay.

## Introduction

Atrial fibrillation (AF) is the most common form of arrhythmia among adults in the United States (US).^1^ More than 3 million people in the US have AF, with this number expected to increase to 7.5 million by 2050.^2^ For AF patients who do not tolerate or are unresponsive to antiarrhythmic drugs, catheter ablation (CA) is indicated.^3^, ^4^, ^5^, ^6^, ^7^, ^8^

CA typically involves follow-up observation for at least 1 overnight stay (ONS) in the outpatient setting^9^; however, a growing body of evidence suggests that it may be feasible and safe to discharge appropriately selected patients home on the same day of their procedure.^10^, ^11^, ^12^, ^13^, ^14^, ^15^, ^16^, ^17^, ^18^ The same-day discharge (SDD) approach has been increasingly utilized.

A recent editorial highlighted the limitations of current evidence supporting the safety and efficacy of CA with SDD.^19^ These limitations include the following: (1) lack of consideration of the impact of transesophageal echocardiography use and body mass index on the procedural outcomes; (2) limited evidence investigating patients with nonparoxysmal AF; and (3) study period not reflective of current practices. Moreover, much of the evidence in the literature is based on single-center experience or small multicenter studies, which limits generalizability and the strength of conclusions. Therefore, we investigated the postprocedural outcomes of SDD vs ONS after elective outpatient CA using a large, real-world US database from 2016 to 2020, comparing complication rate within 30 days and AF recurrence within 1 year.

## Methods

### Study population

This retrospective cohort study examined administrative claims and billing data in the IBM MarketScan® Commercial Claims and Encounters (CCAE) database from January 1, 2016, to June 30, 2020. The CCAE database contains health insurance claims data across inpatient and outpatient services, along with prescription drug and enrollment information for approximately 43 million persons (and their dependents) annually who are covered under employer-sponsored insurance in the US.^20^ As dictated by Title 45 Code of Federal Regulations (45 CFR 46.101(b)(4)) (available at https://www.govinfo.gov/content/pkg/CFR-2011-title45-vol1/pdf/CFR-2011-title45-vol1.pdf), this analysis of the IBM MarketScan database was conducted under an exemption from Institutional Review Board oversight for US-based studies using de-identified healthcare records. The research reported in this paper adhered to guidelines set forth by the Helsinki Declaration as revised in 2013.

Patients who underwent an elective outpatient CA procedure with a primary or secondary diagnosis of AF (*International Classification of Diseases 10*^*th*^ *revision Clinical Modification* [ICD-10-CM] diagnostic codes I48.0, I48.1x, I48.2x, I48.91) between July 1, 2016, and May 31, 2020 were eligible for enrollment. CA procedure was identified using CPT code (93656) and ICD-10 procedure codes (02563ZZ, 02573ZZ, 025K3ZZ, 025L3ZZ, 02583ZZ, 02553ZZ, 025M3ZZ, 025S3ZZ, 025T3ZZ). For the assessment of efficacy outcomes, a subset of patients from the initial cohort who underwent CA procedure between July 1, 2016, and June 30, 2019 were included to ensure a potential 1 year of follow-up after index procedure. Patients were indexed on the first CA for AF if multiple eligible procedures were identified during the identification period. Each cohort was further stratified into SDD and ONS groups. Patients were classified as having SDD after CA if their dates for admission and discharge were the same. They were classified as having ONS if the discharge date was 1 day after the admission date.

**Figure 1:**
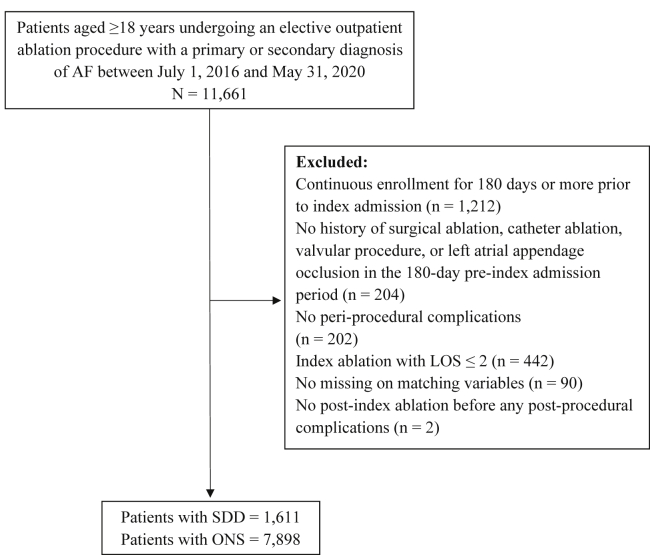
Flow chart of 30-day follow-up cohort inclusion. Patients undergoing an elective outpatient ablation procedure between July 1, 2016, and May 31, 2020 were screened for the inclusion in the 30-day follow-up cohort. AF = atrial fibrillation; LOS = length of stay; ONS = overnight stay; SDD = same-day discharge.

**Figure 2:**
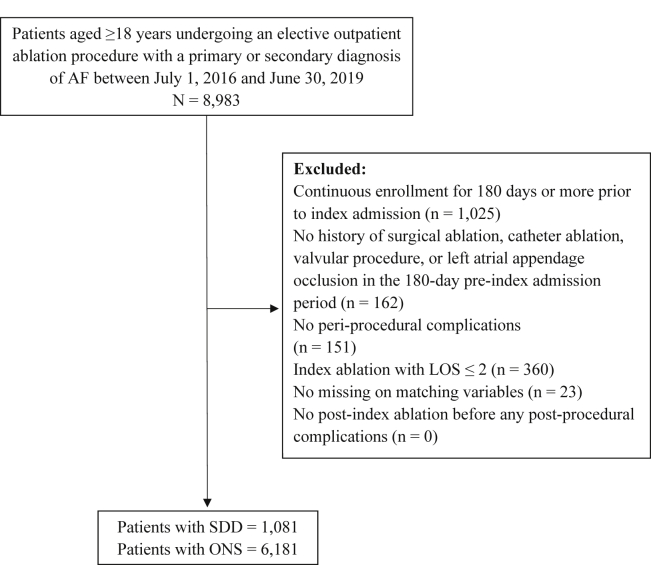
Flow chart of 1-year follow-up cohort inclusion. Patients undergoing an elective outpatient ablation procedure between July 1, 2016, and June 30, 2019 were screened for inclusion in the 1-year follow-up cohort. AF = atrial fibrillation; LOS = length of stay; ONS = overnight stay; SDD = same-day discharge.

To be included in the study, patients had to be ≥18 years of age at the time of index admission and be continuously enrolled for at least 180 days prior to the index admission. Patients were excluded if they (1) had catheter or surgical ablation, valvular procedure, or left atrial appendage occlusion in the 180-day pre–index admission period; and (2) stayed more than 1 day after the index CA procedure. We further excluded patients who had complications during the index CA admission, because patients who had periprocedural complications were more likely to have ONS,^17^ and SDD protocol usually requires a that procedure occurred without complications.^10^^,^^16^^,^^18^ For patients who had any postprocedural complication identified, we examined whether those patients had repeat ablation before the date of complication recorded and within 30 days after the index ablation. If present, we excluded them because the identified postprocedural complication could have been associated with either the index or repeat CA. Figures 1 and 2 depict the study attrition.

### Covariates

Patient demographic information was collected, including age, sex, and insurance type. Patient clinical characteristics in the 180-day pre-index period and during the time of index admission were also collected, including Elixhauser comorbidity score,^21^ CHA₂DS₂-VASc score,^22^ antiarrhythmic drug use, and oral anticoagulant use. Comorbidities were identified using ICD-10-CM diagnosis codes, which are based on primary or secondary diagnosis codes as listed during the pre-index period and index admission. Other covariates were intracardiac echocardiography (ICE) use during the index procedure and region of the hospital/provider.

### Type of AF

We defined patients having paroxysmal AF (PAF) if they had ICD-10-CM diagnosis code for PAF (I48.0) but no other type of AF (I48.1x, I48.2x, I48.91) identified from the claim records on the procedure date. Similarly, persistent AF (PsAF) patients were those with a diagnosis code of I48.1x only on the procedure date.

### Outcomes of interest

The primary safety outcome was the overall complication rate within the 30-day postprocedure period. A composite variable for complications was defined as including any of the following type of complication: myocardial infarction, pericardial complications, cerebrovascular events, vascular access events, respiratory complications, phrenic nerve damage, bleeding complications, systemic inflammatory response syndrome and sepsis, cardiac complications, and acute venous embolism and thrombosis. A breakdown list of each type of complication and the ICD-10-CM/CPT codes used for the identification can be found in Supplemental Table 1. Time-to-event for postprocedure complications, both composite and individual type of complication, were assessed in the 30-day period.

AF recurrence within a year of the procedure was the primary efficacy outcome of interest and was assessed during the 4–12 months post–index ablation. AF recurrence was a composite variable defined as an inpatient visit for AF, cardioversion, and repeat CA. The first occurrence of any component of the composite efficacy outcome was used. Time-to-event for AF recurrence, as well as the component variables, were assessed during the 4–12 months post–index ablation.

### Statistical analysis

To examine and compare outcomes among the two study groups, propensity score matching (PSM) (greedy match algorithm with 0.1 caliper) was used. We also conducted a sensitivity analysis to include the specific year that the CA procedure was performed as one of the covariates in the propensity score matching process. All baseline characteristics were used as matching factors. Standardized mean differences (SMD) for the matching factors were assessed, with differences above 0.10 or below -0.10 considered as a sign of imbalance. Bivariate statistical analysis techniques were used to test for statistically significant differences in the outcomes between the matched two groups.

Patients were censored from statistical analysis if they were lost to follow-up (discontinuous enrollment with a gap of ≥1 day) or if they reached the end of follow-up time (maximum 30 days for complications; maximum 1 year for AF recurrence) without an outcome of interest. Cox proportional hazard model was used to assess differences in study outcomes in the matched cohort. The primary independent variable of interest included in the regression model was day of discharge (SDD vs ONS). Any covariates that emerged as significant (SMD >0.10 or <-0.10) post-matching were adjusted for in the Cox regression analysis. A sub-analysis by type of AF (PAF and PsAF) was performed. In all analyses, a 2-sided *P* < .05 was the threshold by which differences were considered statistically significant. All analyses were conducted using R for Windows, version 4.0.0 (Foundation for Statistical Computing, Vienna, Austria).

## Results

### Thirty-day cohort characteristics

There were 9509 total patients in the CCAE database who met eligibility criteria for the 30-day follow-up cohort. Of these, 16.9% (1611/9509) had SDD and 83.1% (7898/9509) had ONS. No significant difference in mean age was observed among the 2 cohorts (56.1 [standard deviation ±7.68] years in SDD vs. 56.2 [standard deviation ±7.39] years in ONS, *P* = .563). Before PSM, patients in the SDD group had lower percentage of female sex (22.5% in SDD vs 27.4% in ONS, *P* < .001), lower comorbidity burden (26.6% in SDD vs 31.6% in ONS with Elixhauser comorbidity score ≥5, *P* < .001), and lower CHA₂DS₂-VASc score (1.57 [±1.20] in SDD vs 1.70 [±1.20] in ONS, *P* < .001). The use of antiarrhythmic drugs (60.0% vs 69.9%, *P* < .001), oral anticoagulants (78.3% vs 82.3%, *P* < .001), and ICE (91.7% vs 96.0%, *P* < .001) was also significantly lower in the SDD group as compared to the ONS group. In the postmatch 30-day cohort, there were 1610 SDD and 4637 ONS patients. Only 1 patient from the SDD group was dropped after PSM, and all baseline covariates were balanced between SDD and ONS with the absolute values of SMD less than 0.1. A full list of baseline characteristics in the 30-day prematch and postmatch cohorts appears in Table 1.

**Table 1:** Baseline characteristics of prematch and postmatch 30-day follow-up cohort

	Prematch				Postmatch (1:3)			
	SDD	ONS	*P* value†	SMD	SDD	ONS	*P* value†	SMD
	N = 1611	N = 7898			N = 1610	N = 4637		
Age group			.394				.803	
18–49	276 (17.1)	1256 (15.9)		0.033	276 (17.1)	762 (16.4)		0.013
50–59	667 (41.4)	3379 (42.8)		-0.028	667 (41.4)	1941 (41.9)		-0.004
60–69	668 (41.5)	3263 (41.3)		0.003	667 (41.4)	1934 (41.7)		-0.006
Female	363 (22.5)	2161 (27.4)	<.001	0.112	363 (22.5)	1033 (22.3)	.850	0.019
Insurance			.009				.994	
PPO	870 (54.0)	4466 (56.5)		-0.051	870 (54.0)	2535 (54.7)		-0.002
CDHP	181 (11.2)	952 (12.1)		-0.026	181 (11.2)	526 (11.3)		0.000
HMO	166 (10.3)	697 (8.8)		0.050	166 (10.3)	467 (10.1)		0.006
HDHP	151 (9.4)	809 (10.2)		-0.029	151 (9.4)	439 (9.5)		-0.001
POS	122 (7.6)	521 (6.6)		0.038	122 (7.6)	337 (7.3)		0.003
Other/unknown	121 (7.5)	453 (5.7)		0.071	120 (7.5)	333 (7.2)		-0.005
Elixhauser score			<.001				.817	
1–2	492 (30.5)	2071 (26.2)		0.096	492 (30.6)	1378 (29.7)		0.006
3–4	690 (42.8)	3330 (42.2)		0.014	689 (42.8)	2010 (43.3)		-0.011
5+	429 (26.6)	2497 (31.6)		-0.110	429 (26.6)	1249 (26.9)		0.005
CHA₂DS₂-VASc score	1.57 (1.20)	1.70 (1.20)	<.001	-0.112	1.57 (1.20)	1.58 (1.20)	.730	0.003
Sleep apnea	659 (40.9)	3332 (42.2)	.356	-0.026	658 (40.9)	1907 (41.1)	.880	0.001
AAD use	966 (60.0)	5520 (69.9)	<.001	-0.209	966 (60.0)	2868 (61.9)	.199	-0.002
Anticoagulants use	1262 (78.3)	6503 (82.3)	<.001	-0.101	1261 (78.3)	3668 (79.1)	.532	-0.002
ICE use	1478 (91.7)	7586 (96.0)	<.001	-0.181	1478 (91.8)	4353 (93.9)	.005	-0.022
Provider's region			<.001				.450	
Midwest	360 (22.3)	2147 (27.2)		-0.112	360 (22.4)	1080 (23.3)		-0.005
Northeast	283 (17.6)	1005 (12.7)		0.135	283 (17.6)	758 (16.3)		0.010
South	689 (42.8)	3802 (48.1)		-0.108	689 (42.8)	2043 (44.1)		-0.002
West	279 (17.3)	944 (12.0)		0.152	278 (17.3)	756 (16.3)		-0.001

CDHP = consumer-driven health plans; CI = confidence interval; HDHP = high-deductible health plan; HMO = health maintenance organization; HR = hazard ratio; ICE = intracardiac echocardiography; ONS = overnight stay; POS = point of service; PPO = preferred provider organization; SDD = same-day discharge; SMD = standardized mean difference.

† *P* values were calculated from χ^2^ test or *t* test.

### Thirty-day complications

There was no significant difference in composite 30-day postprocedural complications between SDD and ONS patients (2.7% in SDD vs 2.8%, in ONS, hazard ratio [HR] = 0.97, 95% confidence interval [CI] 0.68–1.36, *P* from Cox proportional hazards model = .840). In addition, results from bivariate analysis and Cox proportional hazards model showed no significant differences in any of the individual types of complication investigated. The most common complications were cerebrovascular events (0.7% in SDD vs 0.7% in ONS, HR = 1.02, 95% CI 0.51–2.04, *P* = .948), vascular access events (0.6% in SDD vs 0.6% in ONS, HR = 1.03, 95% CI 0.50–2.12, *P* = .935), respiratory complications (0.6% in SDD vs 0.4% in ONS, HR = 1.52, 95% CI 0.71–3.27, *P* = .285), and pericardial complications (0.6% SDD vs 0.5% ONS, HR = 1.04, 95% CI 0.49–2.23, *P* = .921). Results from the comparison of complication endpoints are listed in Table 2.

**Table 2:** 30-day complication rates in postmatch same-day discharge and overnight stay groups

	Bivariate comparison			Cox regression			
	SDD (%) N = 1610	ONS (%) N = 4637	*P*†	HR	95% CI		*P*‡
Composite complications	43 (2.7)	129 (2.8)	.884	0.965	0.683	1.363	.840
Cerebrovascular events	11 (0.7)	31 (0.7)	1.000	1.023	0.514	2.036	.948
Vascular access events	10 (0.6)	28 (0.6)	1.000	1.030	0.500	2.121	.935
Respiratory complications	10 (0.6)	19 (0.4)	.389	1.518	0.706	3.266	.285
Pericardial complications	9 (0.6)	25 (0.5)	1.000	1.039	0.485	2.227	.921
Myocardial infarction	4 (0.2)	13 (0.3)	1.000	0.886	0.289	2.718	.833
Cardiac complications	4 (0.2)	11 (0.2)	1.000	1.049	0.334	3.294	.935
Sepsis and SIRS	1 (0.1)	13 (0.3)	.197	0.221	0.029	1.692	.146
Acute venous embolism and thrombosis	0 (0.0)	6 (0.1)	.329	0.000	0.000	N/A	.998
Bleeding complications	0 (0.0)	3 (0.1)	.718	0.000	0.000	N/A	.999
Phrenic nerve damage	0 (0.0)	1 (0.0)	1.000	0.000	0.000	N/A	.999

CI = confidence interval; HR = hazard ratio; N/A, not applicable; ONS = overnight stay; SDD = same-day discharge; SIRS = systematic inflammatory response syndrome.

† *P* values were based on χ^2^ test.

‡ *P* values were based on Cox proportional hazards model.

### One-year cohort characteristics

As a subset of the 30-day cohort, there were 7262 total patients in the CCAE database who were identified for the assessment of AF recurrence. Of these, 14.9% (1081/7262) had SDD and 85.1% (6181/7262) had ONS. In the postmatch 1-year cohort, there were 1079 SDD and 3128 ONS patients with balanced baseline covariates between the 2 study groups. A full list of baseline characteristics in the 1-year prematch and postmatch cohorts appears in Table 3.

**Table 3:** Baseline characteristics of postmatch 1-year follow-up cohort

	Prematch				Postmatch (1:3)			
	SDD	ONS	*P* value†	SMD	SDD	ONS	*P* value†	SMD
	N = 1081	N = 6181			N = 1079	N = 3128		
Age group			.437				.917	
18–49	188 (17.4)	1002 (16.2)		0.032	188 (17.4)	528 (16.9)		0.012
50–59	444 (41.1)	2655 (43.0)		-0.038	444 (41.1)	1292 (41.3)		0.002
60–69	449 (41.5)	2524 (40.8)		0.014	447 (41.4)	1308 (41.8)		-0.011
Female	247 (22.8)	1691 (27.4)	.002	0.104	247 (22.9)	739 (23.6)	.653	-0.008
Insurance			.045				1.000	
PPO	579 (53.6)	3539 (57.3)		-0.074	579 (53.7)	1696 (54.2)		0.000
CDHP	133 (12.3)	733 (11.9)		0.014	132 (12.2)	381 (12.2)		0.000
HMO	105 (9.7)	530 (8.6)		0.040	105 (9.7)	296 (9.5)		0.005
HDHP	93 (8.6)	592 (9.6)		-0.034	93 (8.6)	265 (8.5)		0.009
POS	81 (7.5)	379 (6.1)		0.054	81 (7.5)	232 (7.4)		-0.008
Other/Unknown	90 (8.3)	408 (6.6)		0.066	89 (8.2)	258 (8.2)		-0.007
Elixhauser score			<.001				.954	
1–2	331 (30.6)	1680 (27.2)		0.076	330 (30.6)	972 (31.1)		-0.018
3–4	478 (44.2)	2590 (41.9)		0.047	477 (44.2)	1370 (43.8)		0.009
5+	272 (25.2)	1911 (30.9)		-0.128	272 (25.2)	786 (25.1)		0.008
CHA₂DS₂-VASc score	1.58 (1.21)	1.69 (1.20)	.006	-0.090	1.58 (1.21)	1.59 (1.20)	.827	-0.001
Sleep apnea	416 (38.5)	2574 (41.6)	.056	-0.065	415 (38.5)	1186 (37.9)	.778	0.015
AAD use	673 (62.3)	4348 (70.3)	<.001	-0.172	673 (62.4)	1979 (63.3)	.625	0.005
Anticoagulants use	835 (77.2)	5068 (82.0)	<.001	-0.118	834 (77.3)	2441 (78.0)	.642	-0.006
ICE use	977 (90.4)	5925 (95.9)	<.001	-0.218	977 (90.5)	2903 (92.8)	.020	-0.013
Provider's region			<.001				.624	
Midwest	235 (21.7)	1650 (26.7)		-0.116	235 (21.8)	722 (23.1)		-0.015
Northeast	202 (18.7)	783 (12.7)		0.166	201 (18.6)	541 (17.3)		0.004
South	451 (41.7)	2974 (48.1)		-0.129	451 (41.8)	1331 (42.6)		0.003
West	193 (17.9)	774 (12.5)		0.149	192 (17.8)	534 (17.1)		0.009

CDHP = consumer-driven health plans; CI = confidence interval; HDHP = high-deductible health plan; HMO = health maintenance organization; HR = hazard ratio; ICE = intracardiac echocardiography; ONS = overnight stay; POS = point of service; PPO = preferred provider organization; SDD = same-day discharge; SMD = standardized mean difference.

† *P* values were calculated from χ^2^ test or *t* test.

### One-year AF recurrence

Results assessing the AF recurrence rate from bivariate analysis and Cox proportional hazard model are shown in Table 4. There was no significant difference in composite AF recurrence rate during the 1-year follow-up (10.2% in SDD vs 8.8% in ONS, HR = 1.17, 95% CI 0.94–1.46, *P* = .172). Examining efficacy outcomes individually, there were no significant differences in patients’ AF-related inpatient readmission (3.3% in SDD vs 3.2% in ONS, HR = 1.04, 95% CI 0.71–1.52, *P* = .844), electrical cardioversion (3.7% in SDD vs 3.5% in ONS, HR = 1.06, 95% CI 0.74–1.52, *P* = .756), and repeat ablation (6.3% in SDD vs 5.1% in ONS, HR = 1.25, 95% CI 0.94–1.67, *P* = .119).

**Table 4:** One-year atrial fibrillation recurrence rates in postmatch same-day discharge and overnight stay groups

	Bivariate comparison			Cox regression			
	SDD (%) N = 1079	ONS (%) N = 3128	*P*†	HR	95% CI		*P*‡
Composite recurrence	110 (10.2)	275 (8.8)	.188	1.167	0.935	1.455	.172
Inpatient readmission	36 (3.3)	100 (3.2)	.902	1.039	0.710	1.521	.844
Electrical cardioversion	40 (3.7)	109 (3.5)	.806	1.059	0.737	1.522	.756
Repeat ablation	68 (6.3)	158 (5.1)	.135	1.254	0.944	1.666	.119

CI = confidence interval; HR = hazard ratio; ONS = overnight stay; SDD = same-day discharge.

† *P* values were based on χ^2^ test.

‡ *P* values were based on Cox proportional hazards model.

### Sub-analyses for PAF and PsAF

In the sub-analysis, we identified 2573 PAF patients (27.1%; 2573/9509) in the 30-day follow-up cohort and 1913 PAF patients (26.3%; 1913/7262) in the 1-year follow-up cohort. The baseline characteristics for pre- and postmatch cohorts for PAF patients are shown in Supplemental Table 2a and 2c. Among PAF patients, there was no significant difference between SDD and ONS in composite complication rate (2.6% in SDD vs 3.1% in ONS, HR = 0.867, 95% CI 0.455–1.652, *P* = .664, Supplemental Table 2b) and AF recurrence rate (6.2% in SDD vs 6.5% in ONS, HR = 0.974, 95% CI 0.564–1.685, *P* = .926, Supplemental Table 2d). Also, none of the component safety and efficacy outcomes differ between SDD and ONS groups in patients with PAF (Supplemental Table 2b and 2d). There were 878 and 788 patients with PsAF identified in the 30-day and 1-year follow-up cohorts, respectively. The baseline characteristics for pre- and postmatch cohorts for PsAF patients are shown in Supplemental Table 3a and 3c. Similarly, no differences between SDD and ONS were found in all study outcomes, including composite complication rate (2.3% in SDD vs 3.0% in ONS, HR = 0.739, 95% CI 0.205–2.658, *P* = .643, Supplemental Table 3b) and AF recurrence (8.5% in SDD vs 15.0% in ONS, HR = 0.629, 95% CI 0.278–1.421, *P* = .265, Supplemental Table 3d). Other results for component safety and efficacy measures are displayed in Supplemental Table 3b and 3d.

Given that ablation techniques and discharge pathways may have changed over time, we conducted a sensitivity analysis of our outcomes adjusting for the year in which the procedure was performed. There was no difference in outcomes compared to the primary analysis (Supplemental Table 4a–4d).

## Discussion

In this large, commercially insured US cohort, we observed that the postprocedural outcomes, including acute safety and long-term efficacy measures, were similar between SDD and ONS for patients undergoing outpatient CA for AF. We observed nondifferential outcomes of SDD as compared to ONS after matching for patient characteristics, including comorbidity burden, concurrent medications, and use of ICE during the CA procedure. In this study, patients who underwent elective outpatient CA procedure were chosen based on strict criteria, including no cardiovascular procedures within 6 months prior to the CA procedure, no intraprocedural and periprocedural complications during the admission for CA, and being discharged no longer than 1 day after the procedure (not including the day for CA). Given the study design and representative study sample, the findings from our study illustrated that SDD appears to be safe and feasible after CA for AF in carefully selected patients.

The feasibility of SDD has been described in the published literature. Although the selection and discharge criteria vary by study, the success rate of SDD has been suggested to be between 79.1% and 99.2%, among patients who meet the criteria for SDD.^10^^,^^15^, ^16^, ^17^, ^18^ The reasons for not achieving SDD could be varied. A recent chart review of 426 patients following implementation of the SDD strategy after elective outpatient CA for AF or left atrial flutter showed that 50 patients (12%) were not discharged same day. Among these 50 patients, 17 (34%) could not be discharged the same day owing to ablation-related complications, 15 owing to non-ablation-related medical care, and 18 owing to patient preference.^17^ The SDD protocol usually includes procedure on the morning list (end before 2 PM), procedure occurred without complications, no evidence of complications after ambulation, stable hemodynamics, and purse-string suture removed.^10^^,^^16^^,^^18^ To assess the postprocedural outcomes, our study identified a selective cohort of patients without complications on the date of index CA procedure and during the observation period and no consecutive inpatient admission after the CA procedure. We further matched the SDD group with a comparable ONS group using PSM by considering important covariates that could potentially affect the outcomes of interest.

Patient safety should be the most important driver to determine the hospitalization duration after CA procedure. A recent multicenter cohort study by Deyell and colleagues^16^ of 3054 patients with AF undergoing CA reported the complication rate within 30 days after procedure to be 0.37% in 2406 patients with SDD and 0.36% in 551 patients without intraprocedural complication but discharged the day after (*P* = .999). Only the severe complications (death, stroke or embolism, and bleeding), however, were studied in this large cohort study. A few small single-center studies using a broader definition for postprocedural complications, including phrenic nerve paralysis, cardiac tamponade, and vascular complications, showed a 2.3%–3.9% overall postprocedural complication rate with no significant difference in patients with SDD compared to patients with longer hospitalization.^14^^,^^15^^,^^17^ In our study, no significant differences in overall complication rate (or for selected complications) were observed among the SDD and ONS patients undergoing CA for AF.

Though safety outcome comparisons between SDD and ONS patients have been described, limited information is available on long-term efficacy outcomes associated with CA for AF among these two cohorts. In one such study, patients with SDD (7.7%) had significant lower 30-day readmission rate compared to ONS (10.2%) after CA for AF.^16^ Another study investigated the outcomes of a high-throughput AF ablation service (SDD is preferred) within a local noncardiac center compared with matched patients at the regional tertiary cardiac center (regular discharge protocol). This study reported similar efficacy outcomes at 3 month after discharge between the two centers: the postprocedural outcomes including complete resolution of symptoms (54.3% in local vs 54.1% in regional, *P* = 1.00), improvement of symptoms (26.1% in local vs 27.9% in regional, *P* = .90), and redo procedures requested (16.6% vs 17.4%, *P* = 1.00).^23^ Ours is one of the first studies to examine long-term (12-month) efficacy outcomes among SDD and ONS patients. Results from our study suggest no significant difference in repeat ablation (6.3% for SDD vs 5.1% for ONS), electrical cardioversion (3.7% for SDD vs 3.5% for ONS), readmission (3.3% for SDD vs 3.2% for ONS), or composite outcome (10.2% for SDD vs 8.8% for ONS).

Besides studying safety and efficacy profile among the overall AF cohort, our study also examined any differential by AF type (PAF or PsAF). In our sub-analysis by AF type (PAF or PsAF), no significant differences were observed in the safety and efficacy measures. In patients with PAF, the overall postprocedural complication rate was 2.6% in SDD and 3.1% in ONS; and no differences were found in any component complications, such as vascular access events (0.7% in SDD group vs 1.1% in ONS group), cerebrovascular events (0.4% in SDD group vs 0.5% in ONS group), and pericardial complications (0.9% in SDD group vs 0.6% in ONS group). AF recurrence rate seems to be lower in patients with PAF (6.4% of 1078 postmatch PAF cohort) than the overall patients with all type of AF (9.2% of 4207 postmatch PAF cohort), but the rates for any of the efficacy measures were not different between SDD and ONS groups in patients with PAF: readmission 2.9% in SDD vs 1.7% in ONS, electrical cardioversion 1.1% in SDD vs 1.7% in ONS, and repeat ablation 2.9% in SDD vs 4.7% in ONS. In patients with PsAF, 3 patients of 131 in the SDD group (2.3%) and 11 patients of 369 in ONS (3.0%) had postprocedural complications, 2 of 3 patents in SDD and 3 of 11 patients in ONS had respiratory complications (1.5% in SDD vs 0.8% in ONS), and 1 of 3 patients in SDD and 3 of 11 in ONS had myocardial infarction (0.8% in both groups). None of these differences were statistically significant. Postprocedural efficacy measures, such as electrical cardioversion (3.7% in SDD vs 8.4% in ONS) and repeat ablation (4.9% in SDD vs 7.0% in ONS), as well as the composite outcome (8.5% in SDD vs 15.0% in ONS), tend to be higher in the ONS group; however, the differences were not significant. Despite the different disease profile for PAF and PsAF patients, it may be feasible to have SDD in a carefully selected cohort of patients after CA for both these AF types.

Studies have detailed best practices to improve patient comfort and safety with SDD, suggesting standardized approaches to patient pain management and addressing common complications that may arise.^24^ It will be important to investigate optimal discharge criteria to create best practices and guidelines. Appropriate selection of patients to undergo SDD after CA may further ensure the safety of patients prior to implementing SDD protocols.^18^ Various publications have shared their SDD protocols that were used to select appropriate patients. These include baseline factors such as stable, uninterrupted anticoagulation without requirement for bridging; absence of bleeding history, systolic dysfunction, or recent heart failure; patient resides in close proximity to the hospital; and presence of a competent caregiver at home with the patient for at least 24 hours. Additional postprocedure criteria such as absence of evidence of complication or other concern, stable vital signs, and ability to ambulate without pain have been used. Because this was a de-identified administrative dataset, we were not able to identify or evaluate the specific criteria used to determine SDD in the current study. Moving forward, it will be important to investigate optimal discharge criteria to create best practices and guidelines. Enhanced imaging technology (like ICE) and vascular closure methods are likely to play a critical role in SDD protocols. Studies have shown lower occurrence of major complications including cardiac perforation with the use of ICE,^25^ which is critical when considering patient eligibility for SDD. In a recent study by Mohammed and colleagues,^26^ the use of vascular closure devices instead of manual compression among patients undergoing CA was found to be associated with lower length of stay and increase in SDD.

Our study has several limitations. The source population of the CCAE database primarily represents people with employee-sponsored insurance. Therefore, the study results may not be generalizable to patients older than age 65 or individuals with noncommercial insurance. The lack of information on the specific discharge criteria and protocols limits the ability to comment on best practice and to apply these findings in practice. We additionally lacked information on the time of procedure and defined the SDD and ONS cases by using calendar date. As a result, CA procedure followed by a 6-hour observational period would be more likely defined as SDD if it was a day case and defined as ONS if it was performed in the afternoon in our study. Lastly, the database used for the study did not include information on CA procedures, such as procedure duration, type of CA and anesthetic, the experience of the operator or center volume, closure device, and postprocedure care. Differences in those procedure characteristics between study groups, if present, could have introduced confounding owing to their association with the procedural outcomes. In addition, the observational design means that there is likely an indication bias in determining who received SDD that can only be partially overcome by our use of propensity score matching.

## Conclusion

In our analysis of a large, real-world administrative claims dataset, similar safety and efficacy profiles were observed for AF patients undergoing CA with SDD vs ONS. The safety and efficacy profile of patients in SDD and ONS groups were found to be comparable, even when studied by AF type (PAF or PsAF). As providers adjust hospital protocols to better manage and perform CA in both a pandemic and postpandemic era, our results suggest SDD after CA has safety and efficacy outcomes comparable to ONS approach for a carefully selected cohort of patients.

## Acknowledgments

We acknowledge Superior Medical Experts for their assistance with drafting and editing.

## Funding Sources

This study is supported by Johnson and Johnson.

## Disclosures

MF received research support from Johnson and Johnson, Boston Scientific, and Medtronic. MG is a consultant and receives research support from Medtronic and Boston Scientific. LG, KC, RK, and XF are Johnson and Johnson employees.

## Authorship

All authors attest they meet the current ICMJE criteria for authorship.

## Patient Consent

Patient consent is not applicable, as this analysis was conducted using de-identified healthcare records.

## Ethics Statement

The research reported in this paper adhered to guidelines set forth by the Helsinki Declaration as revised in 2013. This analysis was conducted under an exemption from Institutional Review Board oversight for US-based studies using de-identified healthcare records.
