# Supplementary material for: Evaluating outcomes of same-day discharge after catheter ablation for atrial fibrillation in a real-world cohort
Source: Heart Rhythm O2. 2021 Jul 14;2(4):333–40. doi: 10.1016/j.hroo.2021.07.001 (PMC8369301; doi:10.1016/j.hroo.2021.07.001)
Supplement: Supplemental Tables 1-4 [file mmc1.docx]

**Supplementary Table 1. List of catheter ablation related complications**

| **Complication group** | **Complication** |
| --- | --- |
| Respiratory Complications | pleural effusion, pneumothorax, pulmonary insufficiency, acute respiratory failure |
| Cerebrovascular Events | cerebral occlusion or stenosis, hemorrhagic Stroke, iatrogenic stroke, ischemic stroke, precerebral occlusion or stenosis, pulmonary embolism, transient ischemic attack (TIA) |
| Vascular Access Events | arteriovenous fistula, hematoma, hemorrhage, seroma complicating a procedure, lower extremity aneurysm, lower extremity embolism, vascular repair |
| Myocardial Infarction (Acute) | acute myocardial infarction |
| Bleeding Complications | acute post-hemorrhagic anemia, blood transfusion |
| Sepsis and Sirs | sepsis and sirs |
| Phrenic Nerve Damage | diaphragmatic paralysis, nervous system complications (nerve damage) |
| Acute Venous Embolism & Thrombosis | acute venous embolism and thrombosis |
| Cardiac Complication | cardiac complications |
| Pericardial Complications | acute pericarditis, cardiac tamponade, hemopericardium, hemothorax, pericardial drainage procedure, unspecified disease of pericardium (pericardial effusion) |

**Supplementary Table 2a.** Baseline Characteristics of Pre-Match and Post-Match 30-Day Follow-Up Cohort in Patients with Paroxysmal Atrial Fibrillation

|  | **Pre-match** | | | | **Post-match (1:3)** | | | |
| --- | --- | --- | --- | --- | --- | --- | --- | --- |
|  | **SDD** | **ONS** | **p-value**† | **SMD** | **SDD** | **ONS** | **p-value**† | **SMD** |
|  | n = 457 | n = 2116 |  |  | n = 454 | n = 1303 |  |  |
| **Age group** |  |  | 0.567 |  |  |  | 0.844 |  |
| 18-49 | 86 (18.8) | 396 (18.7) |  | 0.003 | 86 (18.9) | 231 (17.7) |  | 0.030 |
| 50-59 | 185 (40.5) | 910 (43.0) |  | -0.051 | 185 (40.7) | 537 (41.2) |  | -0.010 |
| 60-69 | 186 (40.7) | 810 (38.3) |  | 0.050 | 183 (40.3) | 535 (41.1) |  | -0.014 |
| **Female** | 128 (28.0) | 710 (33.6) | 0.025 | 0.120 | 128 (28.2) | 372 (28.5) | 0.933 | 0.008 |
| **Insurance** |  |  | 0.071 |  |  |  | 0.994 | 0.031 |
| PPO | 249 (54.5) | 1152 (54.4) |  | 0.001 | 249 (54.8) | 698 (53.6) |  |  |
| CDHP | 34 (7.4) | 252 (11.9) |  | -0.152 | 34 (7.5) | 109 (8.4) |  | -0.019 |
| HMO | 60 (13.1) | 224 (10.6) |  | 0.079 | 59 (13.0) | 170 (13.0) |  | -0.019 |
| HDHP | 49 (10.7) | 229 (10.8) |  | -0.003 | 48 (10.6) | 142 (10.9) |  | -0.001 |
| POS | 34 (7.4) | 134 (6.3) |  | 0.044 | 33 (7.3) | 95 (7.3) |  | -0.004 |
| Other/Unknown | 31 (6.8) | 125 (5.9) |  | 0.036 | 31 (6.8) | 89 (6.8) |  | -0.009 |
| **Elixhauser score** |  |  | 0.409 |  |  |  | 0.930 |  |
| 1-2 | 149 (32.6) | 700 (33.1) |  | -0.010 | 149 (32.8) | 415 (31.8) |  | 0.025 |
| 3-4 | 208 (45.5) | 900 (42.5) |  | 0.060 | 207 (45.6) | 602 (46.2) |  | -0.017 |
| 5+ | 100 (21.9) | 516 (24.4) |  | -0.059 | 98 (21.6) | 286 (21.9) |  | -0.008 |
| **CHA₂DS₂-VASc score** | 1.52 (1.18) | 1.54 (1.17) | 0.710 | -0.019 | 1.52 (1.18) | 1.50 (1.16) | 0.843 | 0.015 |
| **Sleep apnea** | 192 (42.0) | 786 (37.1) | 0.059 | 0.100 | 190 (41.9) | 545 (41.8) | 1.000 | -0.011 |
| **AAD use** | 276 (60.4) | 1404 (66.4) | 0.018 | -0.124 | 275 (60.6) | 810 (62.2) | 0.586 | -0.017 |
| **Anticoagulants use** | 345 (75.5) | 1696 (80.2) | 0.030 | -0.112 | 344 (75.8) | 1018 (78.1) | 0.332 | -0.038 |
| **ICE use** | 421 (92.1) | 2041 (96.5) | <0.001 | -0.188 | 421 (92.7) | 1238 (95.0) | 0.088 | 0.000 |
| **Provider's region** |  |  | 0.001 |  |  |  | 0.874 |  |
| Midwest | 116 (25.4) | 622 (29.4) |  | -0.090 | 116 (25.6) | 341 (26.2) |  | 0.007 |
| Northeast | 81 (17.7) | 252 (11.9) |  | 0.164 | 79 (17.4) | 206 (15.8) |  | -0.006 |
| South | 188 (41.1) | 978 (46.2) |  | -0.103 | 188 (41.4) | 555 (42.6) |  | -0.002 |
| West | 72 (15.8) | 264 (12.5) |  | 0.094 | 71 (15.6) | 201 (15.4) |  | 0.001 |

† p values were calculated from Chi-squared test or t-test.

CDHP: consumer-driven health plans; CI: confidence interval; HDHP: high deductible health plan; HMO: health maintenance organization; HR: hazard ratio; ICE: intracardiac echocardiography; POS: point of service; PPO: preferred provider organization; SD: standard deviation; SDD: same-day discharge; ONS: overnight stay SMD: standardized mean difference.

**Supplementary Table 2b.** 30-Day Complication Rates in Post-Match SDD and ONS Groups in Patients with Paroxysmal Atrial Fibrillation

|  | **Bivariate comparison** | | | **Cox regression** | | | |
| --- | --- | --- | --- | --- | --- | --- | --- |
|  | **SDD (%)**  n=454 | **ONS (%)**  n=1303 | **p†** | **HR** | **95% CI** | | **p‡** |
| Composite complications | **12 (2.6)** | **40 (3.1)** | **0.763** | **0.867** | **0.455** | **1.652** | **0.664** |
| Cerebrovascular events | 2 (0.4) | 7 (0.5) | 1.000 | 0.822 | 0.171 | 3.958 | 0.807 |
| Vascular access events | 3 (0.7) | 14 (1.1) | 0.619 | 0.616 | 0.177 | 2.143 | 0.446 |
| Respiratory complications | 2 (0.4) | 5 (0.4) | 1.000 | 1.147 | 0.223 | 5.914 | 0.869 |
| Pericardial complications | 4 (0.9) | 8 (0.6) | 0.792 | 1.442 | 0.434 | 4.790 | 0.550 |
| Myocardial infarction | 0 (0.0) | 2 (0.2) | 0.978 | 0.000 | 0.000 | Inf | 0.999 |
| Cardiac complications | 1 (0.2) | 3 (0.2) | 1.000 | 0.960 | 0.100 | 9.226 | 0.972 |
| Sepsis and SIRS | 0 (0.0) | 4 (0.3) | 0.542 | 0.000 | 0.000 | Inf | 0.998 |
| Acute venous embolism & thrombosis | 0 (0.0) | 0 (0.0) | NA | 0.000 | 0.000 | Inf | NA |
| Bleeding complications | 0 (0.0) | 1 (0.1) | 1.000 | 0.000 | 0.000 | Inf | 0.999 |
| Phrenic nerve damage | 0 (0.0) | 4 (0.3) | 0.542 | 0.000 | 0.000 | Inf | 0.998 |

**†** p values were based on Chi-squared test.

**‡** p values were based on Cox proportional-hazards model.

CI: confidence interval; HR: hazard ratio; SDD: same-day discharge; SIRS: systematic inflammatory response syndrome.

**Supplementary Table 2c.** Baseline Characteristics of Post-Match One-Year Follow-Up Cohort in Patients with Paroxysmal Atrial Fibrillation

|  | **Pre-match** | | | | **Post-match (1:3)** | | | |
| --- | --- | --- | --- | --- | --- | --- | --- | --- |
|  | **SDD** | **ONS** | **p value†** | **SMD** | **SDD** | **ONS** | **p value†** | **SMD** |
|  | n = 280 | n = 1633 |  |  | n = 276 | n = 802 |  |  |
| **Age group** |  |  | 0.305 |  |  |  | 0.877 |  |
| 18-49 | 48 (17.1) | 312 (19.1) |  | -0.051 | 48 (17.4) | 138 (17.2) |  | 0.014 |
| 50-59 | 114 (40.7) | 711 (43.5) |  | -0.057 | 113 (40.9) | 342 (42.6) |  | -0.021 |
| 60-69 | 118 (42.1) | 610 (37.4) |  | 0.098 | 115 (41.7) | 322 (40.1) |  | 0.010 |
| **Female** | 80 (28.6) | 541 (33.1) | 0.151 | -0.099 | 79 (28.6) | 241 (30.0) | 0.711 | -0.027 |
| **Insurance** |  |  | 0.653 |  |  |  | 0.976 | 0.011 |
| PPO | 152 (54.3) | 900 (55.1) |  | -0.017 | 149 (54.0) | 432 (53.9) |  |  |
| CDHP | 26 (9.3) | 188 (11.5) |  | -0.073 | 26 (9.4) | 89 (11.1) |  | -0.053 |
| HMO | 30 (10.7) | 166 (10.2) |  | 0.018 | 30 (10.9) | 80 (10.0) |  | 0.030 |
| HDHP | 25 (8.9) | 160 (9.8) |  | -0.030 | 25 (9.1) | 69 (8.6) |  | 0.017 |
| POS | 22 (7.9) | 103 (6.3) |  | 0.060 | 21 (7.6) | 63 (7.9) |  | -0.021 |
| Other/Unknown | 25 (8.9) | 116 (7.1) |  | 0.067 | 25 (9.1) | 69 (8.6) |  | 0.009 |
| **Elixhauser score** |  |  | 0.335 |  |  |  | 0.816 |  |
| 1-2 | 91 (32.5) | 552 (33.8) |  | -0.028 | 89 (32.2) | 270 (33.7) |  | -0.018 |
| 3-4 | 131 (46.8) | 692 (42.4) |  | 0.089 | 130 (47.1) | 360 (44.9) |  | 0.026 |
| 5+ | 58 (20.7) | 389 (23.8) |  | -0.075 | 57 (20.7) | 172 (21.4) |  | -0.010 |
| **CHA₂DS₂-VASc score** | 1.55 (1.21) | 1.52 (1.17) | 0.630 | 0.031 | 1.54 (1.18) | 1.56 (1.19) | 0.786 | -0.030 |
| **Sleep apnea** | 110 (39.3) | 601 (36.8) | 0.467 | 0.051 | 107 (38.8) | 313 (39.0) | 0.996 | -0.011 |
| **AAD use** | 178 (63.6) | 1071 (65.6) | 0.558 | -0.042 | 176 (63.8) | 526 (65.6) | 0.636 | -0.018 |
| **Anticoagulants use** | 201 (71.8) | 1296 (79.4) | 0.006 | -0.177 | 198 (71.7) | 571 (71.2) | 0.925 | 0.035 |
| **ICE use** | 255 (91.1) | 1571 (96.2) | <0.001 | -0.211 | 255 (92.4) | 755 (94.1) | 0.375 | 0.002 |
| **Provider's region** |  |  | 0.011 |  |  |  | 0.896 |  |
| Midwest | 70 (25.0) | 474 (29.0) |  | -0.091 | 70 (25.4) | 203 (25.3) |  | 0.019 |
| Northeast | 52 (18.6) | 204 (12.5) |  | 0.168 | 49 (17.8) | 133 (16.6) |  | 0.000 |
| South | 114 (40.7) | 749 (45.9) |  | -0.104 | 114 (41.3) | 350 (43.6) |  | -0.038 |
| West | 44 (15.7) | 206 (12.6) |  | 0.089 | 43 (15.6) | 116 (14.5) |  | 0.029 |

**†** p-values were calculated from Chi-squared test or t-test.

CDHP: consumer-driven health plans; CI: confidence interval; HDHP: high deductible health plan; HMO: health maintenance organization; HR: hazard ratio; ICE: intracardiac echocardiography; POS: point of service; PPO: preferred provider organization; SD: standard deviation; SDD: same-day discharge; SMD: standardized mean difference.

**Supplementary Table 2d.** One-Year Atrial Fibrillation Recurrence Rates in Post-Match SDD and ONS Groups in Patients with Paroxysmal Atrial Fibrillation

|  | **Bivariate comparison** | | | **Cox regression** | | | | |  |
| --- | --- | --- | --- | --- | --- | --- | --- | --- | --- |
|  | **SDD (%)**  n=276 | **ONS (%)**  n=802 | **p†** | | **HR** | **95% CI** | | **p‡** | |
| Composite recurrence | 17 (6.2) | 52 (6.5) | 0.962 | | 0.974 | 0.564 | 1.685 | 0.926 | |
| Inpatient readmission | 8 (2.9) | 14 (1.7) | 0.357 | | 1.726 | 0.724 | 4.115 | 0.218 | |
| Electrical cardioversion | 3 (1.1) | 14 (1.7) | 0.633 | | 0.633 | 0.182 | 2.203 | 0.472 | |
| Repeat ablation | 8 (2.9) | 38 (4.7) | 0.258 | | 0.619 | 0.289 | 1.327 | 0.218 | |

**†** p values were based on Chi-squared test.

**‡** p values were based on Cox proportional-hazards model.

CI: confidence interval; HR: hazard ratio; SDD: same-day discharge.

**Supplementary Table 3a.** Baseline Characteristics of Pre-Match and Post-Match 30-Day Follow-Up Cohort in Patients with Persistent Atrial Fibrillation

|  | **Pre-match** | | | | **Post-match (1:3)** | | | |
| --- | --- | --- | --- | --- | --- | --- | --- | --- |
|  | **SDD** | **ONS** | **p value†** | **SMD** | **SDD** | **ONS** | **p value†** | **SMD** |
|  | n = 136 | n = 742 |  |  | n = 131 | n = 369 |  |  |
| **Age group** |  |  | 0.606 |  |  |  | 0.938 |  |
| 18-49 | 15 (11.0) | 75 (10.1) |  | 0.030 | 15 (11.5) | 40 (10.8) |  | 0.017 |
| 50-59 | 56 (41.2) | 340 (45.8) |  | -0.094 | 53 (40.5) | 145 (39.3) |  | 0.057 |
| 60-69 | 65 (47.8) | 327 (44.1) |  | 0.075 | 63 (48.1) | 184 (49.9) |  | -0.066 |
| **Female** | 16 (11.8) | 153 (20.6) | 0.022 | -0.242 | 16 (12.2) | 44 (11.9) | 1.000 | 0.028 |
| **Insurance** |  |  | 0.066 |  |  |  | 0.923 |  |
| PPO | 77 (56.6) | 435 (58.6) |  | -0.041 | 77 (58.8) | 222 (60.2) |  | -0.021 |
| CDHP | 18 (13.2) | 84 (11.3) |  | 0.058 | 17 (13.0) | 44 (11.9) |  | 0.019 |
| HMO | 16 (11.8) | 60 (8.1) |  | 0.123 | 14 (10.7) | 38 (10.3) |  | 0.030 |
| HDHP | 5 (3.7) | 79 (10.6) |  | -0.273 | 5 (3.8) | 22 (6.0) |  | -0.070 |
| POS | 8 (5.9) | 44 (5.9) |  | -0.002 | 8 (6.1) | 21 (5.7) |  | 0.022 |
| Other/Unknown | 12 (8.8) | 40 (5.4) |  | 0.134 | 10 (7.6) | 22 (6.0) |  | 0.030 |
| **Elixhauser score** |  |  | 0.056 |  |  |  | 0.965 |  |
| 1-2 | 29 (21.3) | 133 (17.9) |  | 0.086 | 27 (20.6) | 80 (21.7) |  | -0.019 |
| 3-4 | 66 (48.5) | 304 (41.0) |  | 0.152 | 64 (48.9) | 179 (48.5) |  | -0.031 |
| 5+ | 41 (30.1) | 305 (41.1) |  | -0.230 | 40 (30.5) | 110 (29.8) |  | 0.048 |
| **CHA₂DS₂-VASc score** | 1.75 (1.15) | 1.96 (1.22) | 0.065 | -0.176 | 1.78 (1.15) | 1.74 (1.19) | 0.729 | 0.033 |
| **Sleep apnea** | 73 (53.7) | 381 (51.3) | 0.685 | 0.047 | 71 (54.2) | 199 (53.9) | 1.000 | -0.005 |
| **AAD use** | 84 (61.8) | 552 (74.4) | 0.003 | -0.273 | 82 (62.6) | 247 (66.9) | 0.428 | -0.041 |
| **Anticoagulants use** | 117 (86.0) | 645 (86.9) | 0.884 | -0.026 | 112 (85.5) | 316 (85.6) | 1.000 | 0.007 |
| **ICE use** | 123 (90.4) | 710 (95.7) | 0.019 | -0.208 | 123 (93.9) | 348 (94.3) | 1.000 | 0.030 |
| **Provider's region** |  |  | 0.500 |  |  |  | 0.961 |  |
| Midwest | 43 (31.6) | 242 (32.6) |  | -0.021 | 41 (31.3) | 123 (33.3) |  | -0.035 |
| Northeast | 21 (15.4) | 105 (14.2) |  | 0.036 | 19 (14.5) | 48 (13.0) |  | 0.039 |
| South | 52 (38.2) | 316 (42.6) |  | -0.089 | 52 (39.7) | 144 (39.0) |  | 0.029 |
| West | 20 (14.7) | 79 (10.6) |  | 0.122 | 19 (14.5) | 54 (14.6) |  | -0.034 |

**†** p values were calculated from Chi-squared test or t-test.

CDHP: consumer-driven health plans; CI: confidence interval; HDHP: high deductible health plan; HMO: health maintenance organization; HR: hazard ratio; ICE: intracardiac echocardiography; POS: point of service; PPO: preferred provider organization; SD: standard deviation; SDD: same-day discharge; ONS: overnight stay SMD: standardized mean difference.

**Supplementary Table 3b.** 30-Day Complication Rates in Post-Match SDD and ONS Groups in Patients with Paroxysmal Atrial Fibrillation

|  | **Bivariate comparison** | | | **Cox regression** | | | |
| --- | --- | --- | --- | --- | --- | --- | --- |
|  | **SDD (%)**  n=131 | **ONS (%)**  n=369 | **p†** | **HR** | **95% CI** | | **p‡** |
| Composite complications | **3 (2.3)** | **11 (3.0)** | **0.918** | **0.739** | **0.205** | **2.658** | **0.643** |
| Cerebrovascular events | 0 (0.0) | 2 (0.5) | 0.969 | 0.000 | 0.000 | Inf | 0.999 |
| Vascular access events | 0 (0.0) | 1 (0.3) | 1.000 | 0.000 | 0.000 | Inf | 0.999 |
| Respiratory complications | 2 (1.5) | 3 (0.8) | 0.846 | 1.775 | 0.296 | 10.659 | 0.530 |
| Pericardial complications | 0 (0.0) | 2 (0.5) | 0.969 | 0.000 | 0.000 | Inf | 0.999 |
| Myocardial infarction | 1 (0.8) | 3 (0.8) | 1.000 | 0.979 | 0.101 | 9.532 | 0.985 |
| Cardiac complications | 0 (0.0) | 2 (0.5) | 0.969 | 0.000 | 0.000 | Inf | 0.999 |
| Sepsis and SIRS | 0 (0.0) | 1 (0.3) | 1.000 | 0.000 | 0.000 | Inf | 0.999 |
| Acute venous embolism & thrombosis | 0 (0.0) | 0 (0.0) | NA | NA | NA | NA | NA |
| Bleeding complications | 0 (0.0) | 0 (0.0) | NA | NA | NA | NA | NA |
| Phrenic nerve damage | 0 (0.0) | 1 (0.3) | 1.000 | 0.000 | 0.000 | Inf | 0.999 |

**†** p values were based on Chi-squared test.

**‡** p values were based on Cox proportional-hazards model.

CI: confidence interval; HR: hazard ratio; SDD: same-day discharge; SIRS: systematic inflammatory response syndrome.

**Supplementary Table 3c.** Baseline Characteristics of Post-Match One-Year Follow-Up Cohort in Patients with Persistent Atrial Fibrillation

|  | **Pre-match** | | | | **Post-match (1:3)** | | | |
| --- | --- | --- | --- | --- | --- | --- | --- | --- |
|  | **SDD** | **ONS** | **p-value†** | **SMD** | **SDD** | **ONS** | **p-value†** | **SMD** |
|  | n = 85 | n = 554 |  |  | n = 82 | n = 227 |  |  |
| **Age group** |  |  | 0.457 |  |  |  | 0.838 |  |
| 18-49 | 9 (10.6) | 56 (10.1) |  | 0.016 | 9 (11.0) | 30 (13.2) |  | -0.073 |
| 50-59 | 34 (40.0) | 261 (47.1) |  | -0.144 | 34 (41.5) | 88 (38.8) |  | 0.107 |
| 60-69 | 42 (49.4) | 237 (42.8) |  | 0.133 | 39 (47.6) | 109 (48.0) |  | -0.061 |
| **Female** | 10 (11.8) | 120 (21.7) | 0.049 | -0.268 | 10 (12.2) | 36 (15.9) | 0.537 | -0.088 |
| **Insurance** |  |  | 0.480 |  |  |  | 0.960 |  |
| PPO | 47 (55.3) | 334 (60.3) |  | -0.101 | 46 (56.1) | 132 (58.1) |  | -0.049 |
| CDHP | 11 (12.9) | 60 (10.8) |  | 0.065 | 11 (13.4) | 29 (12.8) |  | 0.038 |
| HMO | 10 (11.8) | 52 (9.4) |  | 0.077 | 9 (11.0) | 19 (8.4) |  | 0.093 |
| HDHP | 4 (4.7) | 51 (9.2) |  | -0.178 | 4 (4.9) | 14 (6.2) |  | -0.032 |
| POS | 6 (7.1) | 28 (5.1) |  | 0.084 | 5 (6.1) | 17 (7.5) |  | -0.102 |
| Other/Unknown | 7 (8.2) | 29 (5.2) |  | 0.120 | 7 (8.5) | 16 (7.0) |  | 0.065 |
| **Elixhauser score** |  |  | 0.135 |  |  |  | 0.939 |  |
| 1-2 | 18 (21.2) | 100 (18.1) |  | 0.079 | 17 (20.7) | 45 (19.8) |  | 0.056 |
| 3-4 | 42 (49.4) | 228 (41.2) |  | 0.166 | 40 (48.8) | 108 (47.6) |  | -0.045 |
| 5+ | 25 (29.4) | 226 (40.8) |  | -0.240 | 25 (30.5) | 74 (32.6) |  | -0.000 |
| **CHA₂DS₂-VASc score** | 1.87 (1.21) | 1.96 (1.20) | 0.529 | -0.073 | 1.89 (1.21) | 1.91 (1.14) | 0.908 | -0.062 |
| **Sleep apnea** | 40 (47.1) | 285 (51.4) | 0.524 | -0.088 | 38 (46.3) | 109 (48.0) | 0.895 | 0.016 |
| **AAD use** | 59 (69.4) | 417 (75.3) | 0.308 | -0.131 | 56 (68.3) | 166 (73.1) | 0.489 | -0.105 |
| **Anticoagulants use** | 72 (84.7) | 477 (86.1) | 0.860 | -0.040 | 70 (85.4) | 198 (87.2) | 0.814 | -0.081 |
| **ICE use** | 74 (87.1) | 528 (95.3) | 0.005 | -0.294 | 73 (89.0) | 205 (90.3) | 0.907 | 0.065 |
| **Provider's region** |  |  | 0.244 |  |  |  | 0.970 |  |
| Midwest | 30 (35.3) | 169 (30.5) |  | 0.102 | 28 (34.1) | 84 (37.0) |  | -0.022 |
| Northeast | 14 (16.5) | 85 (15.3) |  | 0.031 | 14 (17.1) | 36 (15.9) |  | 0.006 |
| South | 27 (31.8) | 236 (42.6) |  | -0.226 | 27 (32.9) | 71 (31.3) |  | 0.038 |
| West | 14 (16.5) | 64 (11.6) |  | 0.142 | 13 (15.9) | 36 (15.9) |  | -0.029 |

**†** p-values were calculated from Chi-squared test or t-test.

CDHP: consumer-driven health plans; CI: confidence interval; HDHP: high deductible health plan; HMO: health maintenance organization; HR: hazard ratio; ICE: intracardiac echocardiography; POS: point of service; PPO: preferred provider organization; SD: standard deviation; SDD: same-day discharge; SMD: standardized mean difference.

**Supplementary Table 3d.** One-Year Atrial Fibrillation Recurrence Rates in Post-Match SDD and ONS Groups in Patients with Paroxysmal Atrial Fibrillation

|  | **Bivariate comparison** | | | | **Cox regression** | | | |
| --- | --- | --- | --- | --- | --- | --- | --- | --- |
|  | **SDD (%)**  n=82 | **ONS (%)**  n=227 | **p†** | **HR** | | **95% CI** | | **p‡** |
| Composite recurrence | 7 (8.5) | 34 (15.0) | 0.199 | 0.629 | | 0.278 | 1.421 | 0.265 |
| Inpatient readmission | 2 (2.4) | 9 (4.0) | 0.771 | 0.701 | | 0.151 | 3.253 | 0.650 |
| Electrical cardioversion | 3 (3.7) | 19 (8.4) | 0.241 | 0.482 | | 0.142 | 1.634 | 0.241 |
| Repeat ablation | 4 (4.9) | 16 (7.0) | 0.672 | 0.788 | | 0.263 | 2.366 | 0.672 |

**†** p values were based on Chi-squared test.

**‡** p values were based on Cox proportional-hazards model.

CI: confidence interval; HR: hazard ratio; SDD: same-day discharge.

**Supplementary Table 4a.** Baseline Characteristics of Pre-Match and Post-Match 30-Day Follow-Up Cohort Further Adjusting for Year of Procedure

|  | **Pre-match** | | | | **Post-match (1:3)** | | | |
| --- | --- | --- | --- | --- | --- | --- | --- | --- |
|  | **SDD** | **ONS** | **p-value**† | **SMD** | **SDD** | **ONS** | **p-value**† | **SMD** |
|  | n = 1611 | n = 7898 |  |  | n = 1605 | n = 4553 |  |  |
| **Age group** |  |  | 0.394 |  |  |  | 0.961 |  |
| 18-49 | 276 (17.1) | 1256 (15.9) |  | 0.033 | 273 (17.0) | 778 (17.1) |  | -0.015 |
| 50-59 | 667 (41.4) | 3379 (42.8) |  | -0.028 | 666 (41.5) | 1904 (41.8) |  | 0.002 |
| 60-69 | 668 (41.5) | 3263 (41.3) |  | 0.003 | 666 (41.5) | 1871 (41.1) |  | 0.009 |
| **Female** | 363 (22.5) | 2161 (27.4) | <0.001 | -0.112 | 363 (22.6) | 1055 (23.2) | 0.675 | 0.001 |
| **Insurance** |  |  | 0.009 |  |  |  | 0.888 |  |
| PPO | 870 (54.0) | 4466 (56.5) |  | -0.051 | 867 (54.0) | 2535 (55.7) |  |  |
| CDHP | 181 (11.2) | 952 (12.1) |  | -0.026 | 181 (11.3) | 507 (11.1) |  | -0.023 |
| HMO | 166 (10.3) | 697 (8.8) |  | 0.050 | 164 (10.2) | 451 ( 9.9) |  | 0.008 |
| HDHP | 151 (9.4) | 809 (10.2) |  | -0.029 | 150 ( 9.3) | 412 ( 9.0) |  | 0.003 |
| POS | 122 (7.6) | 521 (6.6) |  | 0.038 | 122 ( 7.6) | 315 ( 6.9) |  | 0.016 |
| Other/Unknown | 121 (7.5) | 453 (5.7) |  | 0.071 | 121 ( 7.5) | 333 ( 7.3) |  | 0.018 |
| **Elixhauser score** |  |  | <0.001 |  |  |  | 0.703 |  |
| 1-2 | 492 (30.5) | 2071 (26.2) |  | 0.096 | 487 (30.3) | 1331 (29.2) |  | 0.003 |
| 3-4 | 690 (42.8) | 3330 (42.2) |  | 0.014 | 690 (43.0) | 1986 (43.6) |  | -0.005 |
| 5+ | 429 (26.6) | 2497 (31.6) |  | -0.110 | 428 (26.7) | 1236 (27.1) |  | 0.002 |
| **CHA₂DS₂-VASc score** | 1.57 (1.20) | 1.70 (1.20) | <0.001 | -0.112 | 1.57 (1.20) | 1.59 (1.19) | 0.524 | 0.001 |
| **Sleep apnea** | 659 (40.9) | 3332 (42.2) | 0.356 | -0.026 | 656 (40.9) | 1842 (40.5) | 0.793 | 0.015 |
| **AAD use** | 966 (60.0) | 5520 (69.9) | <0.001 | -0.209 | 966 (60.2) | 2825 (62.0) | 0.198 | -0.002 |
| **Anticoagulants use** | 1262 (78.3) | 6503 (82.3) | <0.001 | -0.101 | 1257 (78.3) | 3589 (78.8) | 0.694 | -0.000 |
| **ICE use** | 1478 (91.7) | 7586 (96.0) | <0.001 | -0.181 | 1475 (91.9) | 4269 (93.8) | 0.012 | -0.014 |
| **Provider's region** |  |  | <0.001 |  |  |  | 0.334 |  |
| Midwest | 360 (22.3) | 2147 (27.2) |  | -0.112 | 360 (22.4) | 1072 (23.5) |  | -0.007 |
| Northeast | 283 (17.6) | 1005 (12.7) |  | 0.135 | 280 (17.4) | 773 (17.0) |  | -0.011 |
| South | 689 (42.8) | 3802 (48.1) |  | -0.108 | 688 (42.9) | 2002 (44.0) |  | -0.000 |
| West | 279 (17.3) | 944 (12.0) |  | 0.152 | 277 (17.3) | 706 (15.5) |  | 0.020 |
| **Year of procedure** |  |  | <0.001 |  |  |  | 0.280 |  |
| 2016 | 172 (10.7) | 1137 (14.4) |  | -0.113 | 172 (10.7) | 512 (11.2) |  | -0.002 |
| 2017 | 292 (18.1) | 1943 (24.6) |  | -0.158 | 292 (18.2) | 864 (19.0) |  | -0.002 |
| 2018 | 392 (24.3) | 2053 (26.0) |  | -0.038 | 392 (24.4) | 1148 (25.2) |  | -0.000 |
| 2019 | 558 (34.6) | 2270 (28.7) |  | 0.127 | 557 (34.7) | 1571 (34.5) |  | -0.000 |
| 2020 | 197 (12.2) | 495 ( 6.3) |  | 0.207 | 192 (12.0) | 458 (10.1) |  | 0.006 |

† p values were calculated from Chi-squared test or t-test.

CDHP: consumer-driven health plans; CI: confidence interval; HDHP: high deductible health plan; HMO: health maintenance organization; HR: hazard ratio; ICE: intracardiac echocardiography; POS: point of service; PPO: preferred provider organization; SD: standard deviation; SDD: same-day discharge; ONS: overnight stay SMD: standardized mean difference.

**Supplementary Table 4b.** 30-Day Complication Rates in Post-Match SDD and ONS Further Adjusting for Year of Procedure

|  | **Bivariate comparison** | | | **Cox regression** | | | |
| --- | --- | --- | --- | --- | --- | --- | --- |
|  | **SDD (%)**  n=1605 | **ONS (%)**  n=4553 | **p†** | **HR** | **95% CI** | | **p‡** |
| Composite complications | **43 (2.7)** | **113 (2.5)** | **0.734** | **1.085** | **0.764** | **1.542** | **0.647** |
| Cerebrovascular events | 11 (0.7) | 17 (0.4) | 0.167 | 1.840 | 0.862 | 3.929 | 0.115 |
| Vascular access events | 10 (0.6) | 20 (0.4) | 0.483 | 1.422 | 0.666 | 3.039 | 0.363 |
| Respiratory complications | 10 (0.6) | 21 (0.5) | 0.560 | 1.353 | 0.637 | 2.873 | 0.431 |
| Pericardial complications | 9 (0.6) | 28 (0.6) | 0.957 | 0.914 | 0.431 | 1.937 | 0.814 |
| Myocardial infarction | 4 (0.2) | 13 (0.3) | 1.000 | 0.874 | 0.285 | 2.679 | 0.813 |
| Cardiac complications | 4 (0.2) | 7 (0.2) | 0.663 | 1.624 | 0.475 | 5.547 | 0.439 |
| Sepsis and SIRS | 1 (0.1) | 13 (0.3) | 0.190 | 0.218 | 0.029 | 1.667 | 0.142 |
| Acute venous embolism & thrombosis | 0 (0.0) | 4 (0.1) | 0.536 | 0.000 | 0.000 | Inf | 0.998 |
| Bleeding complications | 0 (0.0) | 5 (0.1) | 0.413 | 0.000 | 0.000 | Inf | 0.998 |
| Phrenic nerve damage | 0 (0.0) | 2 (0.0) | 0.973 | 0.000 | 0.000 | Inf | 0.999 |

**†** p values were based on Chi-squared test.

**‡** p values were based on Cox proportional-hazards model.

CI: confidence interval; HR: hazard ratio; SDD: same-day discharge; SIRS: systematic inflammatory response syndrome.

**Supplementary Table 4c.** Baseline Characteristics of Post-Match One-Year Follow-Up Cohort Further Adjusting for Year of Procedure

|  | **Pre-match** | | | | **Post-match (1:3)** | | | |
| --- | --- | --- | --- | --- | --- | --- | --- | --- |
|  | **SDD** | **ONS** | **p-value†** | **SMD** | **SDD** | **ONS** | **p-value†** | **SMD** |
|  | n = 1081 | n = 6181 |  |  | n = 1079 | n = 3147 |  |  |
| **Age group** |  |  | 0.437 |  |  |  | 0.939 |  |
| 18-49 | 188 (17.4) | 1002 (16.2) |  | 0.032 | 188 (17.4) | 540 (17.2) |  | 0.007 |
| 50-59 | 444 (41.1) | 2655 (43.0) |  | -0.038 | 444 (41.1) | 1314 (41.8) |  | -0.008 |
| 60-69 | 449 (41.5) | 2524 (40.8) |  | 0.014 | 447 (41.4) | 1293 (41.1) |  | 0.003 |
| **Female** | 247 (22.8) | 1691 (27.4) | 0.002 | 0.104 | 247 (22.9) | 725 (23.0) | 0.955 | 0.007 |
| **Insurance** |  |  | 0.045 |  |  |  | 0.965 |  |
| PPO | 579 (53.6) | 3539 (57.3) |  | -0.074 | 579 (53.7) | 1675 (53.2) |  | 0.014 |
| CDHP | 133 (12.3) | 733 (11.9) |  | 0.014 | 132 (12.2) | 384 (12.2) |  | 0.003 |
| HMO | 105 (9.7) | 530 (8.6) |  | 0.040 | 105 ( 9.7) | 303 ( 9.6) |  | 0.001 |
| HDHP | 93 (8.6) | 592 (9.6) |  | -0.034 | 93 ( 8.6) | 296 ( 9.4) |  | -0.021 |
| POS | 81 (7.5) | 379 (6.1) |  | 0.054 | 80 ( 7.4) | 217 ( 6.9) |  | 0.009 |
| Other/Unknown | 90 (8.3) | 408 (6.6) |  | 0.066 | 90 ( 8.3) | 272 ( 8.6) |  | -0.017 |
| **Elixhauser score** |  |  | <0.001 |  |  |  | 0.974 |  |
| 1-2 | 331 (30.6) | 1680 (27.2) |  | 0.076 | 330 (30.6) | 955 (30.3) |  | 0.001 |
| 3-4 | 478 (44.2) | 2590 (41.9) |  | 0.047 | 477 (44.2) | 1388 (44.1) |  | 0.003 |
| 5+ | 272 (25.2) | 1911 (30.9) |  | -0.128 | 272 (25.2) | 804 (25.5) |  | -0.004 |
| **CHA₂DS₂-VASc score** | 1.58 (1.21) | 1.69 (1.20) | 0.006 | -0.090 | 1.58 (1.21) | 1.59 (1.19) | 0.717 | -0.008 |
| **Sleep apnea** | 416 (38.5) | 2574 (41.6) | 0.056 | -0.065 | 415 (38.5) | 1206 (38.3) | 0.964 | 0.005 |
| **AAD use** | 673 (62.3) | 4348 (70.3) | <0.001 | -0.172 | 673 (62.4) | 1976 (62.8) | 0.835 | 0.005 |
| **Anticoagulants use** | 835 (77.2) | 5068 (82.0) | <0.001 | -0.118 | 835 (77.4) | 2471 (78.5) | 0.462 | -0.025 |
| **ICE use** | 977 (90.4) | 5925 (95.9) | <0.001 | -0.218 | 977 (90.5) | 2925 (92.9) | 0.013 | -0.018 |
| **Provider's region** |  |  | <0.001 |  |  |  | 0.813 |  |
| Midwest | 235 (21.7) | 1650 (26.7) |  | -0.116 | 235 (21.8) | 713 (22.7) |  | -0.007 |
| Northeast | 202 (18.7) | 783 (12.7) |  | 0.166 | 200 (18.5) | 551 (17.5) |  | 0.001 |
| South | 451 (41.7) | 2974 (48.1) |  | -0.129 | 451 (41.8) | 1336 (42.5) |  | 0.000 |
| West | 193 (17.9) | 774 (12.5) |  | 0.149 | 193 (17.9) | 547 (17.4) |  | 0.007 |
| **Year of procedure** |  |  | <0.001 |  |  |  | 0.772 |  |
| 2016 | 175 (16.2) | 1148 (18.6) |  | -0.063 | 175 (16.2) | 537 (17.1) |  | -0.015 |
| 2017 | 294 (27.2) | 1963 (31.8) |  | -0.100 | 293 (27.2) | 817 (26.0) |  | 0.031 |
| 2018 | 396 (36.6) | 2060 (33.3) |  | 0.069 | 395 (36.6) | 1182 (37.6) |  | -0.019 |
| 2019 | 216 (20.0) | 1010 (16.3) |  | 0.095 | 216 (20.0) | 611 (19.4) |  | 0.002 |

**†** p-values were calculated from Chi-squared test or t-test.

CDHP: consumer-driven health plans; CI: confidence interval; HDHP: high deductible health plan; HMO: health maintenance organization; HR: hazard ratio; ICE: intracardiac echocardiography; POS: point of service; PPO: preferred provider organization; SD: standard deviation; SDD: same-day discharge; SMD: standardized mean difference.

**Supplementary Table 4d.** One-Year Atrial Fibrillation Recurrence Rates in Post-Match SDD and ONS Groups Further Adjusting for Year of Procedure

|  | **Bivariate comparison** | | | **Cox regression** | | | |
| --- | --- | --- | --- | --- | --- | --- | --- |
|  | **SDD (%)**  n=1079 | **ONS (%)**  n=3147 | **p†** | **HR** | **95% CI** | | **p‡** |
| Composite recurrence | 110 (10.2) | 286 ( 9.1) | 0.310 | 1.142 | 0.916 | 1.423 | 0.237 |
| Inpatient readmission | 36 ( 3.3) | 102 ( 3.2) | 0.958 | 1.037 | 0.709 | 1.517 | 0.850 |
| Electrical cardioversion | 40 ( 3.7) | 116 ( 3.7) | 1.000 | 1.012 | 0.706 | 1.449 | 0.949 |
| Repeat ablation | 68 ( 6.3) | 168 ( 5.3) | 0.266 | 1.200 | 0.905 | 1.591 | 0.204 |

**†** p values were based on Chi-squared test.

**‡** p values were based on Cox proportional-hazards model.

CI: confidence interval; HR: hazard ratio; SDD: same-day discharge.
